# Thoughts at the end of the term

**Published:** 2012-03-05

**Authors:** F Popa

**Affiliations:** Carol Davila University of Medicine and PharmacyRomania

My second term as Rector of „Carol Davila” University of Medicine and Pharmacy will come to an end in March. 

The change in the Leadership of the University has already taken place through the vote of the entire academic community, as required by the law. 

At present, until the new Leadership installs and starts taking actions, I still have time to tell you a few thoughts. 

“Carol Davila” University of Medicine and Pharmacy in Bucharest has remained the same reservoir of important personalities of the medical world, even when we talk about the Medical Department of the Romanian Academy, the Academy of Medical Sciences, the Academy of Scientists, different specialty commissions in decision or legislative institutions, and last but not least, the Medical Profile Institutes and the top medical units in Romania. 

The enumeration can be regarded as a pride, when talking about tradition, but also as an extremely serious and difficult obligation. 

The time we are living has proved to be difficult, loaded with conflicts and attempts of finding, through reforms, new ways of putting the human kind back on its feet, by creating a new form of its existence. 

Globally speaking, among the other priorities we can also find the health problem, its protection and improvement representing an extremely important and interesting issue. 

It is obvious that we cannot separate the Health Sector from the Medical Education at all the levels, and, in this context, the medical school in Bucharest, with all its facilitations, has an essential role. 

The process of educating the young generations of doctors and nurses, the research, both fundamental and explanatory, by the development of the working cult, of solidarity and honor, remain the main role of the school.

Analyzing, with a lot of responsibility, the evolution in time of the medical school in Bucharest, we can appreciate its progress in its existence of almost 160 years. 

The moderate optimism can represent a grant for a future development and an ascending trend in the evolution of the University. 

The present school is a link in an institutional chain which it can make more efficient, obviously in the use of the society. 

The way we are now convinced that the expression *Nihil sine deo* has a recognized truth, the same we are sure that nothing can be solid and efficient without the school and its development. 

The next steps will have to be guided decidedly towards the modernization and efficiency of the education, towards the growth of its quality, always being aware of the things that happen around us, whether we are talking about the European space or the space that goes beyond the ocean. 

The access and promotion in school must answer exclusively to the criteria of quality and character. 

The large base, but most importantly the top of the pyramid must be built with patience and responsibility. 

The actual state of the department, especially after the implementation of the new education law, must be analyzed carefully. 

The danger of restricting the educational bases, or, on the contrary, their dilution through inadequate promotions, still exists. 

There are traditional and fundamental disciplines for the education and research, which must be developed and strengthened, the way the obligation of identifying new directions, which should improve the medical education, will have to be rapidly implemented. 

The new concepts of the free circulation of people and professors, the lack of discrimination of any kind, the correct appreciation of the youth, will represent subjects for reflection, delicate and difficult decisions for the new Leadership of the University. 

The corporatist system, which we will have to accept, whether we want it or not, will always bring in the discussion the cost-efficiency report, which will not be ignored and obvious, and, it will not mean that such a slip is a proof of intelligence. 

All these thoughts and obviously, many more which could appear will have to trigger resorts which have not been used yet, in order to make up a valuable Leadership of the University. 

The traditional ways will have to define their professional peaks, and the new ones will have to identify valuable young people who will open and impose them. 

I believe that this way the University will reward the work of our predecessors and it will be lead and guided towards the international recognition that we all wish for. 

